# Alternative hosts of banana bunchy top virus in the Philippines and the first evidence of seed transmission of BBTV

**DOI:** 10.3389/fpls.2024.1467331

**Published:** 2024-11-27

**Authors:** Nicole Angelee P. Mendoza, Jay-Vee S. Mendoza, John E. Thomas, Fe M. Dela Cueva

**Affiliations:** ^1^ National Crop Protection Center, College of Agriculture and Food Science, University of the Philippines Los Baños, Los Baños, Laguna, Philippines; ^2^ Plant Pathology Laboratory, Institute of Plant Breeding, College of Agriculture and Food Science, University of the Philippines Los Baños, Los Baños, Laguna, Philippines; ^3^ Queensland Alliance for Agriculture and Food Innovation, The University of Queensland, St. Lucia, QLD, Australia

**Keywords:** alternative host, banana bunchy top virus, ornamentals, *Pentalonia*, seed transmission

## Abstract

Banana bunchy top disease is caused by *banana bunchy top virus* (BBTV). BBTV is transmitted locally by aphids (*Pentalonia* spp.), but the long-distance spread is through the movement of infected planting materials. This study investigated potential alternative hosts of BBTV in ornamental *Musa* and related species in the *Zingiberales* in the Philippines. Artificial inoculation of BBTV, molecular detection and transmission assay were used to evaluate 15 plant test species. The potential for seed transmission of BBTV through *Canna indica* seeds was also investigated. Seed samples were validated and quantified for BBTV presence using molecular tools, and then grown for transmission assay. Typical symptoms of BBTV in bananas, including dark green streak on the midrib and petiole and rosetting were observed on inoculated *Musa coccinea* (banana blossom), *M. velutina* (velutina), *M laterita*. (bronze banana) and *Canna indica* (Bandera Espanola). PCR assays confirmed BBTV infection in these symptomatic test plants, as well as in *Curcuma longa* (turmeric) which exhibited large chlorotic blotches on the leaf. BBTV was detected from both seeds and germinated seedlings of artificially inoculated and field-collected *C. indica* samples. This study identified *M. laterita* as a new host of BBTV. The susceptibility to BBTV of *M. coccinea, M. velutina*, *C. indica*, and *C. longa* was also confirmed. The study also provided the first evidence of seed transmission of BBTV. *C indica* is an ornamental plant popularly used for landscaping in the Philippines and seeds were shown to be an efficient mode of transmission of the virus with rates up to 34%. The discovery of natural infection in ornamental plants and seeds poses a risk to the banana industry and responsible propagation and appropriate quarantine protocols must be implemented.

## 1 Introduction

Banana bunchy top disease (BBTD), caused by the *banana bunchy top virus* (BBTV), stands as the most economically important viral disease affecting bananas (*Musa* spp.). First recorded in Fiji in 1880, the virus was later studied in detail in the 1920s and 1930s, revealing banana aphids as vectors (Magee, 1927). BBTV has historically devastated banana cropping areas in countries like Australia, Malawi, Pakistan, and India, and its spread persists, as evidenced by recent cases in East Africa (Shimwela et al., 2022; Kesavamoorth, 1980; Thomas et al., 1994a; Ocimati et al., 2021). In the Philippines, BBTV previously devastated banana cultivar ‘Lakatan’ (AAA) bananas in Luzon (Molina et al., 2009) and now threatens banana cv. ‘Saba’ (BBB/ABB) in CALABARZON region. In 2021, cv. Saba farms in Batangas were affected by BBTD (Mendoza et al., 2021), which is particularly concerning given cv. Saba’s known tolerance to the virus. The virus is a member of the species *Babuvirus musae*, genus *Babuvirus* in the family *Nanoviridae*, and has a genome comprising six circular ssDNA components, each separately encapsidated in a 17-19 nm diameter isometric virion (Thomas et al., 2021). These components comprise DNA-R (master Rep), DNA-S (coat protein), DNA-C (cell cycle link protein), DNA-M (movement protein). DNA-N (nuclear shuttle protein) and DNA-U3 encoding a protein of unknown function. Some isolates also contain autonomously replicating, independently, encapsidated alphasatellites, which may act by depressing viral replication and also possibly diverting antiviral RNAi components away from the host virus (Guyot et al., 2022).

Symptoms of BBTD in bananas include leaf marginal chlorosis, dark green streaks on the petiole, distinctive dark green dot and dash flecks on leaves, and a ‘bunchy appearance’ marked by narrow leaves and erect growth as the disease progresses (Thomas et al., 1994b; Magnaye and Valmayor, 1995; Hooks et al., 2008). The exclusive vectors of BBTV are two aphid species: *Pentalonia nigronervosa* (banana aphid) and *P. caladii* (cardamom aphid) (Watanabe et al., 2013; Thomas, 2008; Foottit et al., 2010). Although the banana aphid can carry the virus throughout its lifespan, it cannot transmit it to its offspring (Magee, 1940; Hu et al., 1996).

Reported hosts of BBTV mainly belong to the *Musaceae* family and a few related species, including *M. balbisiana, M. jackeyi, M. acuminata, M. textilis, M. coccinea, M. velutina*, and *Ensete ventricosum* (Wardlaw, 1961; Magee, 1927; Magee, 1948; Thomas and Dietzgen, 1991; Espino et al., 1993; Eusebio and Bajet, 1994; Furuya et al., 2003). In closely related families in the *Zingiberales*, *Alpinia zerumbet*, *A. galangal*, *C. longa* (*Zingiberaceae*), *C. indica* (*Cannaceae*), and *Heliconia* spp. (*Heliconiaceae*) (Pinili et al., 2013; Hamim et al., 2017; Rahayuniati et al., 2021) are reported as alternative hosts of BBTV. Additionally, in the order *Alismetales*, *C. esculenta* (family *Aracaceae*) is identified as an alternative host of BBTV (Pinili et al., 2013). Recognizing alternative hosts is crucial, as they can act as reservoirs and sources of inoculum for the virus, necessitating their inclusion in mitigation efforts against BBTD. Neglecting these hosts may lead to long-distance spread through the unrestricted transfer of infected planting material. Managing BBTV spread is challenging, as infected banana plants typically do not recover. Besides insect transmission, no other modes of transmission for BBTV have been reported, and mechanical transmission using the virus sap is deemed not possible (Thomas et al., 1994b). This study specifically aimed to investigate the potential of ornamental *Musa* and related species in the Philippines to serve as alternative hosts for BBTV.

## 2 Materials and methods

### 2.1 Evaluation of *Musa* and related species against BBTV

For artificial inoculation of BBTV, families from *Zingiberales*, three ornamental species belonging to Musaceae (*M. laterita, M. coccinea* and *M. velutina*), six from Zingiberaceae (*Curcuma longa*, *Hedychium coronarium*, *Etlingera elatior*, *Alpinia purpurata*, *Alpinia luteocarpa* and *Adelmeria gigantifolia*), three from *Cannaceae* (*Canna compacta*, *Canna flacida*, *Canna indica*) and one each from *Heliconiaceae* (*Heliconia* sp.), *Costacea* (*Costus afer*), and *Alismetales* (*Araceae* - *Caladium bicolor*), were evaluated in the study. Two-month-old, PCR-confirmed BBTV-free cv. Lakatan banana plants were used as susceptible control test plants. Twelve replicates were employed for each species.

A standard transmission protocol for BBTV by (Thomas and Dietzgen, 1991) was followed with a few modifications. The BBTV isolate and aphid clone of Dela Cueva et al (Dela Cueva et al., 2023). were employed in the inoculation trial. The artificial inoculation process comprised the following steps: 1) Starvation of *P. nigronervosa* for 4h, 2) 18-24h acquisition access period (AAP) by letting the aphids feed on the BBTV-infected plant and 3) 24-30h inoculation access period (IAP) on test plants. BBTV-inoculated test plants were maintained in an insect-proof greenhouse and regularly observed for symptom development. Ten tissue culture-derived plantlets of cv. Lakatan were also inoculated with BBTV to serve as a susceptible control. The results of transmission were analyzed through regular visual evaluation and PCR assays two months after inoculation and thereafter as symptoms developed.

#### 2.1.2 Molecular detection of BBTV

BBTV detection was performed through PCR assays using extracted DNA. Leaf samples (0.3g) were extracted using Doyle and Doyle’s (Doyle and Doyle, 1987) modified CTAB extraction method (1987). The PCR cocktail contained 1× PCR buffer (Invitrogen, USA), 2 mM MgCl_2_ (Invitrogen, USA), 0.2 mM dNTPs (Invitrogen, USA), 0.2 µM of the forward (BBT1, 3’-CTCGTCATGTGCAAGGTTATGTCG-5’) and reverse (BBT2, -5’ GAAGTTCTCCAGCTATTCATCGCC-3’) primers (Thomson and Dietzgen, 1995) 1 U Taq DNA Polymerase (Invitrogen, USA), 1 µl extracted DNA and DEPC-water to a final volume of 15 µl. PCR assays were performed following cycling conditions: 94°C for 3 min, 35 cycles of 94°C for 1 min, 56°C for 30 s and 72°C for 1 min, and a final 5 min at 72°C. PCR products were resolved by gel electrophoresis in 1.2% agarose gels in 0.5× TAE buffer and were visualized in Molecular Imager^®^ Gel Doc™ XR+ Imaging System.

#### 2.1.3 Transmission assay

Symptomatic test plants were back-indexed to banana cv. Lakatan using the same transmission procedure as described above. Twenty aphids were used per cv. Lakatan plantlet with three replicates per virus source. The result of transmission was analyzed through PCR assays two months after inoculation.

### 2.2 Validation of the presence of BBTV in seeds and seed transmission in *C. indica*

#### 2.2.1 Detection of BBTV from *C. indica* seed samples

BBTV-infected *C. indica* was collected beside an infected banana plant along a roadside at Calauan, Laguna, Philippines (14°08’34.5”N 121°18’42.0”) and labeled as LAG01. The seed samples of LAG01 were analyzed in parallel with harvested seeds from artificially infected *C. indica* plants (IPB01 and IPB04). DNA from 0.3g seed samples was extracted using modified CTAB DNA extraction (Doyle and Doyle, 1987) and used as a template in PCR. The presence of BBTV was detected through the PCR protocol mentioned above.

#### 2.2.2 qPCR detection of BBTV from seed endosperm and embryo

Virus concentrations in the embryo and endosperm of IPB01 were quantified using a SYBR green-based qPCR assay (Mendoza et al., in press)^1^. The embryo was separated from the endosperm using sterile forceps and a scalpel. Seed detection trials were repeated using three individual seeds as replicates. The qPCR mix contained 1× Supermix, 0.1mM each of forward and reverses primer, 1 µl of DNA template, and DEPC to volume up to 10 µl. Quantification cycles (Cq values) were compared using ANOVA in the Rstudio program.

#### 2.2.3 Growing-out test

The *C. indica* seeds were allowed to germinate in seedbeds inside an insect-proof cage with sterile soil and coir dust at a ratio of 1:3. Germinated seedlings were transplanted into individual pots for BBTV detection. Leaf samples of about 0.3g per seedling were collected one month after transplanting and used for DNA extraction. The percent seed transmission was calculated based on the number of seedlings that were positive to the virus over the total number of seedlings tested.

#### 2.2.4 Transmission assay

To determine whether BBTV positive *C. indica* seedlings could serve as an inoculum source for the spread of BBTV, back inoculation to bananas using the standard BBTV transmission protocol (Thomas and Dietzgen, 1991) was conducted. Infected seedlings, IPB01-2 and LAG01-7 served as virus sources for this trial. A total of 900 aviruliferous *P. nigronervosa* were starved for 4 h. Then, aphids were divided to IPB01-2 and LAG01-7 seedlings for an acquisition access feeding period (AAP) with 450 aphids each. After the 24-h AAP, 20 viruliferous aphids were transferred to each of 15 virus-free banana cv. Lakatan test plants for inoculation access period (IAP). The remaining 150 viruliferous aphids were collected for PCR analysis to confirm the acquisition of BBTV by the aphids. Virus-free banana cv. Lakatan plants served as the control. After the IAP, plants were sprayed with insecticide to eliminate the aphids. All plants were maintained inside an insect-proof cage and regularly observed for symptom expression.

## 3 Results

### 3.1 Evaluation of *Musa* and related species for BBTV susceptibility

**Figure 1:**
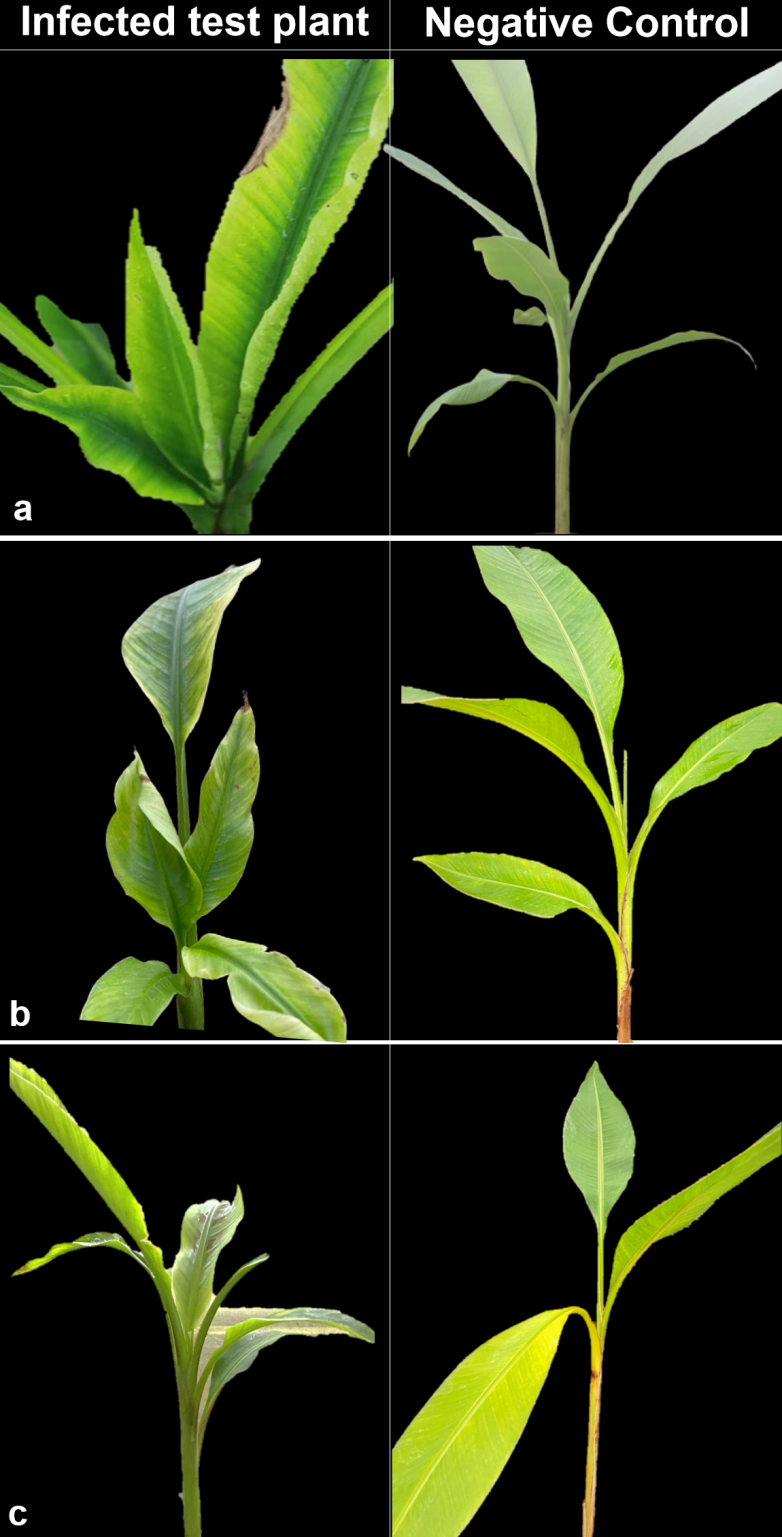
Ornamental *Musa coccinea* **(A)**, *M. velutina* **(B)**, and *M. laterita* **(C)** showing bunchy top symptoms (left) with their negative control (right).

**Figure 2:**
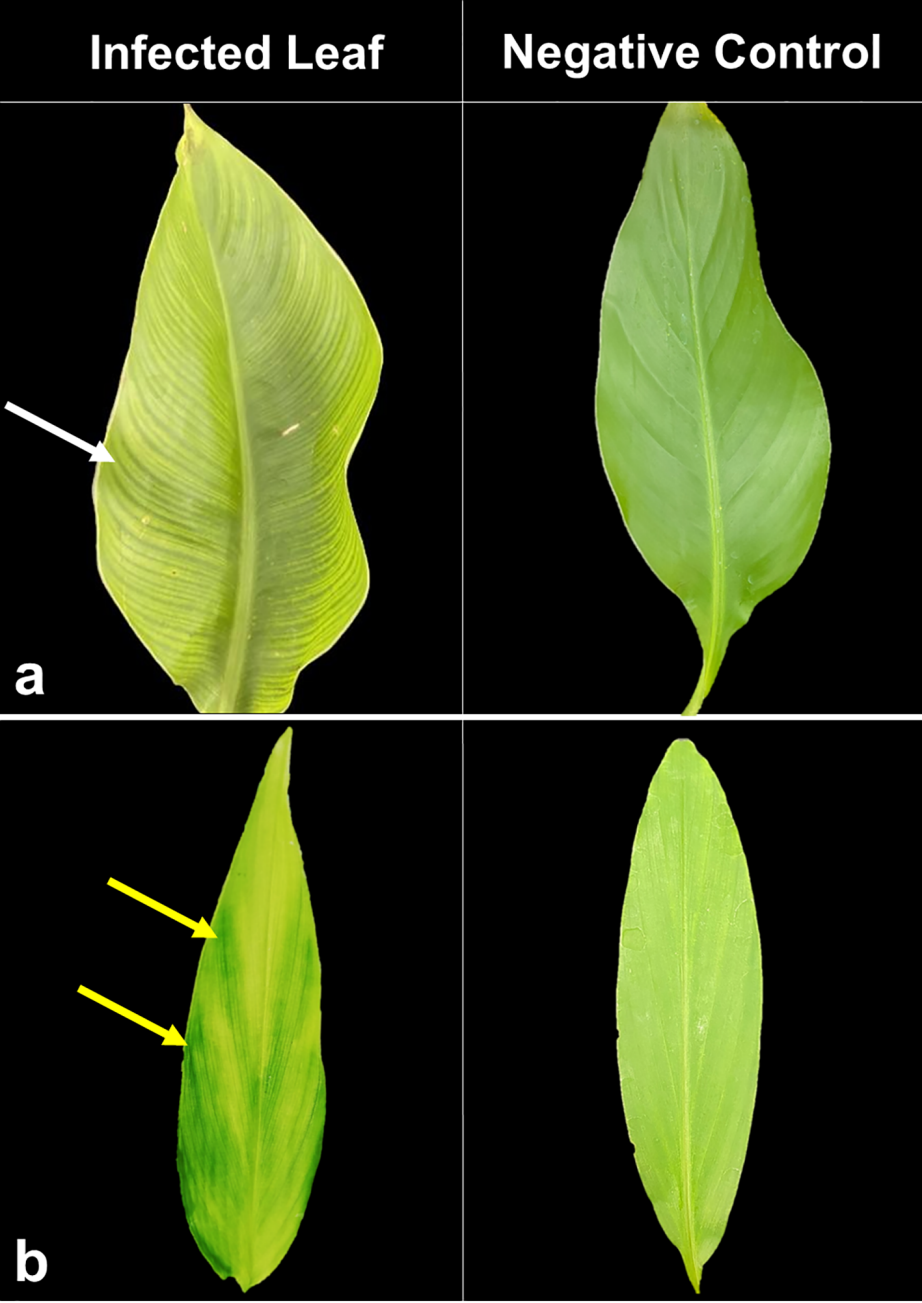
BBTV-infected leaf samples of *C. indica* **(A)** displaying interveinal chlorosis (left) and large chlorotic blotches on *C. longa* **(B)** with negative control on the right.

**Figure 3:**
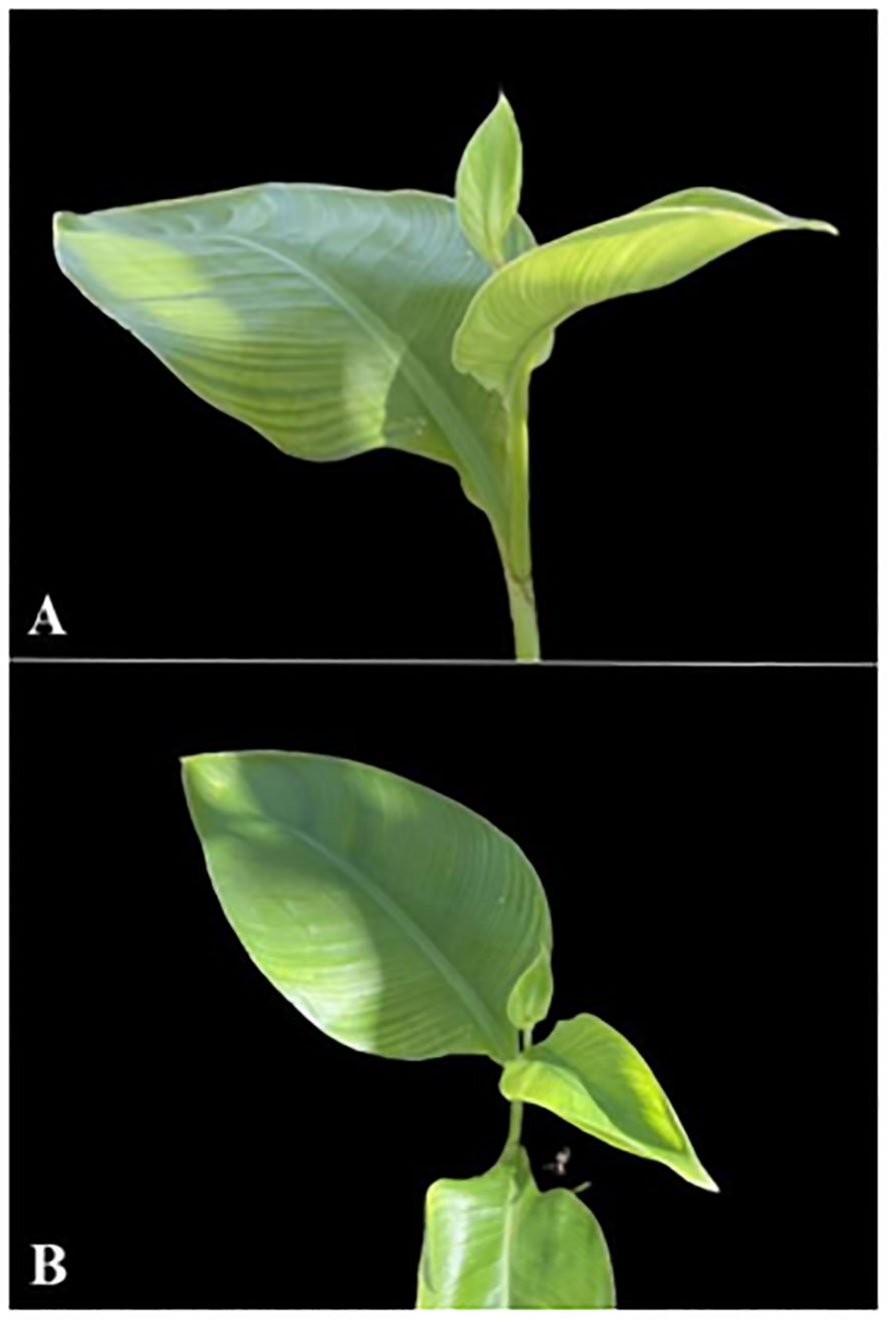
Symptoms of BBTV in *C. indica* IPB01: **(A)** reduction leaf lamina width and petiole length; **(B)** deformed leaf.

Symptoms of BBTD including chlorosis, dark green streaks symptoms along the minor leaf veins, and bunchy leaf appearance were observed in *Musaceae* test plants (**Figure 1**). In *M. velutina*, marginal chlorosis was initially recorded at week four from inoculation. The earliest expression of symptoms in *M. laterita* was observed five weeks after inoculation with chlorosis and green streaks. In the 24^th^ week, a bunchy-top appearance was observed in *M. coccinea* (**Figure 1**). Clearing of leaf veins was observed in leaves of *C. indica* at 8 months after inoculation (**Figure 2**), and size reduction of the youngest leaf and interveinal chlorosis at 15 months (**Figure 3**). Meanwhile, *C. longa* had large chlorotic blotches. No symptoms were observed in any test plant of *Heliconia* sp., *C. bicolor*, *H. coronarium*, *E. elatior*, *A. purpurata*, *A. luteocarpa*, *A. gigantifolia*, *C. compacta*, *C. flacida*, and *C. afer* (**Table 1**).

**Table 1:** Reaction of ornamental *Musa* and related species artificially aphid inoculated with BBTV.

No.	Scientific name	Common Name	Symptoms	Susceptibility to BBTV
1	*Musa coccinea*	Banana blossom	Morse code, dot-dash flecks, onset of bunchy top	+
2	*Musa velutina*	Velutina	Morse code, pre-opening of youngest leaf	+
3	*Musa laterita*	Banana Bronze	Stunted, morse code, dot-dash flecks, j-hook	+
4	*Canna indica*	Bandera Espanola	J-hook and dark green streak	+
5	*Curcuma longa*	Turmeric	Chlorosis on leaves	+
6	*Hedychium coronarium*	Camia	No symptoms	–
7	*Caladium bicolor*	Heart of Jesus	Dark green mosaic-like pattern	–
8	*Heliconia* sp.	Yellow Heliconia	Chlorosis, variegated leaves	–
9	*Canna flacida*	Golden canna	No symptoms	–
10	*Canna compacta*	Compacta	No symptoms	–
11	*Etlingera elatior*	Red torch	No symptoms	–
12	*Alpinia purpurata*	Red ginger	No symptoms	–
13	*Adelmeria gigantifolia*	Gigantifolia	No symptoms	–
14	*Alpinia luteocarpa*	Black ginger	No symptoms	–
15	*Costus afer*	Green wax	No symptoms	–

PCR assays confirmed the infection of BBTV in all symptomatic test plants of *M. coccinea*, *M. velutina* and *M. laterita*. The incidence of BBTV was highest in *M. velutina* with 48%, close to *M*. *laterita* with 31% and 25% in *M. coccinea* based on three repeated trials. Among the other species, only *C. indica* and *C. longa* were positive for BBTV with 38% and 7% recorded incidence, respectively. All other species were negative for BBTV based on PCR assays. There was a 100% infection rate recorded in the positive control cv. Lakatan.

Transmission from PCR-positive symptomatic test species of *M. velutina*, *M. laterita*, and *C. indica* resulted in an infection rate of 100% in banana cv. Lakatan, while from symptomatic *C. longa* the infection rate in cv. Lakatan was 33%. Back-inoculation to control cv. Lakatan resulted in typical BBTV symptoms and infection was confirmed by PCR.

### 3.2 Validation of the presence of BBTV in seeds and seed transmission in *C. indica*

**Figure 4:**
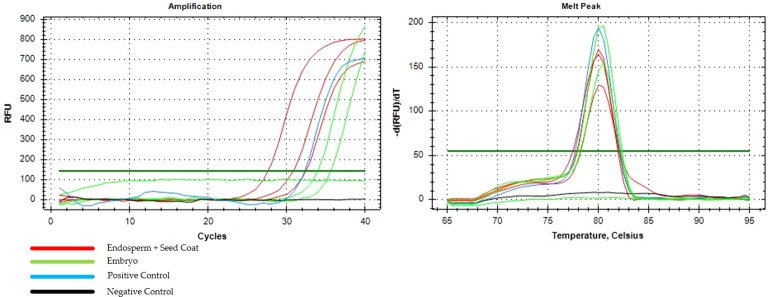
Amplification curves and melt peak graph from the qPCR detection assay of endosperm + seed coat (red) and embryo (green) of *C. indica* seeds.

Eight months after inoculation, *C. indica* IPB01 exhibited symptoms associated with BBTV such as curling of leaf margin and mild interveinal chlorosis. As inoculated plants matured, severe symptoms of BBTV such as reduction of leaf lamina and petiole, bonsai-like symptoms of emerging leaves, vein clearing, and bunchy appearance were noted (**Figure 3**). Despite severe BBTV symptoms, the inoculated plants flowered and produced seeds. End-point PCR detection confirmed the presence of BBTV in *C. indica* IPB01 test plants and seeds. LAG01 plants also yielded positive results when tested using PCR indicating the presence of BBTV. In qPCR of endosperm and embryo, amplification curves and melt peaks confirmed the presence of BBTV in the endosperm+seed coat (3/3) and embryo (2/3) of seeds from IPB01 (**Figure 4**). Cq values from endosperm+seed coat and embryo samples were not significantly different using ANOVA (p=0.102).

The remaining seeds also produced positive transmission results in grow-out tests. The recorded seed germination rates for IPB01 and LAG01 were 32% and 82%, respectively. The PCR results showed that 16/58 (28%) of IPB01 seedlings were positive for BBTV and 11/32 (34%) were positive for LAG-01 one month after germination. BBTV-positive seedlings only showed chlorotic blotches at this time and did not display the symptoms present in the mature parent plants such as shortening of petiole and dark green streaks.

**Figure 5:**
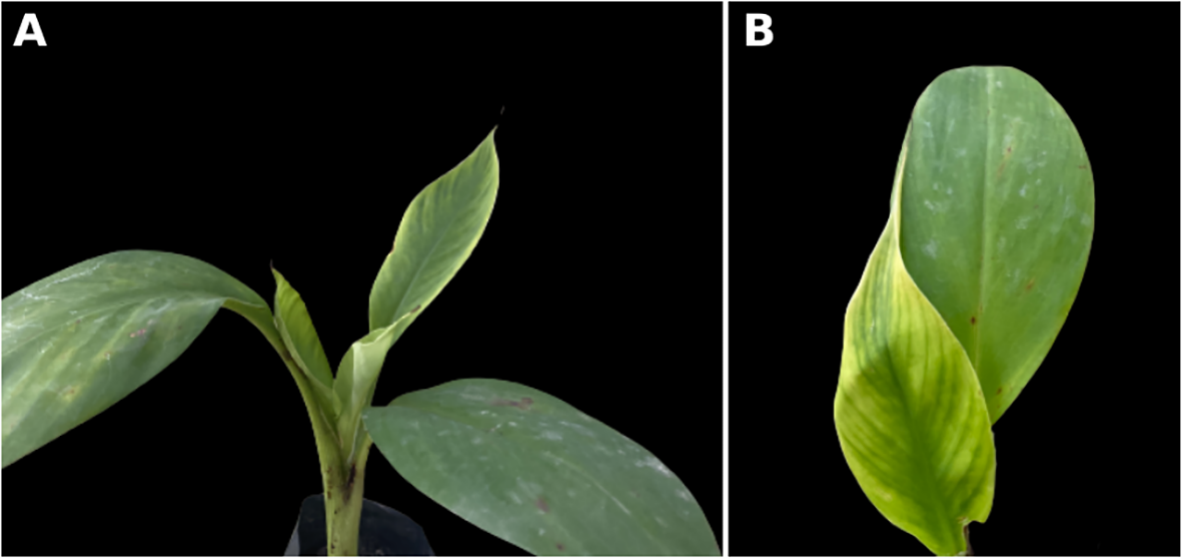
Result of back inoculation test of BBTV from LAG01-7 to banana cv. Lakatan: **(A)** bunchy appearance and **(B)** size reduction of leaf lamina and interveinal chlorosis.

In the transmission test, the PCR assay of 150 aphids each from IPB01-2 and LAG01-7 confirmed the successful acquisition of BBTV by the aphids. Back-indexing of PCR-positive seedlings LAG01-7 and IPB01-2 resulted in 100% infection and typical BBTD symptoms in susceptible control cv. Lakatan. Inoculated cv. Lakatan from virus source LAG01-7 was first to show bunchy top as shown in **Figure 5**. The presence of BBTV in all inoculated cv. Lakatan plants was confirmed by PCR. Uninoculated control plants remained virus-free.

## 4 Discussion

Alternative hosts aid in the spread and survival of pathogens. Reported alternative hosts of BBTV belong to the Zingiberales families *Musaceae*, *Cannaceae*, *Zingiberaceae*, and *Heliconiaceae* and also the family *Araceae* in the *Alismetales* (Thomas, 2024). These families have a diverse set of species that are yet to be tested. Hence, this study evaluated common ornamentals in the Philippines predominantly from the order *Zingiberales* and also from the order *Alismetales*.

The study confirmed the susceptibility of *M. coccinea* to BBTV as reported by Thomas (2019) and also recently in Indonesia of *M. velutina* (Thomas and Dietzgen, 1991; Rahayuniati et al., 2021). On the other hand, this is the first report of *M*. *laterita* (bronze banana) as a new alternative host of BBTV. The presence of BBTV in *C. indica* and *C. longa* test species corroborates the findings of Pinili et al (Pinili et al., 2013; Su et al., 1993). and Rahayuniati et al (Rahayuniati et al., 2021), respectively. *C. longa* is a root crop producing rhizomes commonly known as turmeric. *Canna indica* is a known alternative host of BBTV (Su et al., 1993; Eusebio and Bajet, 1994) and has common names including wild canna lily, Bandera Espanola, edible canna, and Indian shot. It is a perennial herb propagated using seeds and rhizomes. Ornamentals *M. velutina*, *M*. *coccinea*, *M*. *laterita*, *C. indica*, and *C. longa* are widely cultivated in the Philippines as landscape plants. These plants are purchased both online and in physical markets and their distribution could result in the long-distance spread of BBTV-infected planting materials within the Philippines through unregulated transfer. The successful aphid transmission of BBTV from infected *C. indica* to banana cv. Lakatan proves that these plants may act as sources of inoculum for banana in the presence of the banana aphid. This report of alternative hosts will benefit the mitigation efforts in this country against BBTV.

In the present work, *Etlingera elatior, Alpinia purpurata, Adelmeria gigantifolia, Alpinia luteocarpa (Zingiberaceae), Caladium bicolor (Araneae), Heliconia* sp. *(Heliconiaceae), Canna flacida, Canna compacta (Cannaceae)*, and *Costus afer (Costaceae)* appeared to be non-hosts of BBTV in the Philippines. In a report by Su et al (Su et al., 1993), *H. coronarium* is also claimed as a non-host of BBTV. In other reports, BBTV was detected in *Alpinia* species including *A. zerumbet* (Pinili et al., 2013), *A. purpurata* (Wong et al., 2019) and *A. galangal* (Rahayuniati et al., 2021). Natural infection by BBTV in *Heliconia* sp. collected around banana-growing areas was recently reported in Hawaii (Hamim et al., 2017). However, samples of *A. purpurata* and *Heliconia* sp. in this study were negative after multiple rounds of screening. The reason for these discrepancies is not clear, but it seems that the research indicating the individual species as host or non-host was soundly conducted. This situation may result from the particular variety of plant used, the particular isolate of the virus used, or differences in the efficiency of transmission and host preferences of the two known aphid vectors, *P. caladii* and *P. nigronervosa*. Many earlier studies were conducted before these aphids were recognized as two distinct species, so it is unclear which of the two were used.

This wide range of alternative hosts seems to be encompassed within the host ranges of its oligophagous vectors, *P. nigronervosa* and *P. caladii* including mainly members of the *Musaceae, Cannaceae, Araceae, Zingiberaceae*, and *Heliconiaceae* (Foottit et al., 2010; Bagariang et al., 2019). Generally, *P. nigronervosa* favors *Musa* hosts and *P. caladii* the non-*Musa* hosts though there is some overlap (Foottit et al., 2010; Bagariang et al., 2019). This situation opens the possibility for BBTV to be transmitted between these hosts, depending in part on feeding preferences of the two aphid species and the range of co-located plant species in an area.

The BBTV genome itself may play a role in the virus’ ability to infect a variety of hosts, though no specific sequence differences have yet been identified. Several mechanisms may contribute to host adaptability and overall viral fitness. BBTV possesses a multipartite genome, with each of its genome components encapsidated in a separate particle. These components can be expressed independently. This genome organization may provide a selective advantage allowing the virus to rapidly adapt to new hosts and environmental conditions (Foottit et al., 2010; Bagariang et al., 2019; Bashir et al., 2022). Additionally, BBTV has the ability to recombine both inter- and intra-component, enhancing its genetic diversity and evolutionary potential (Fu et al., 2009; Stainton et al., 2015).

Seed transmission of BBTV was an unexpected discovery. There are currently no records of seed transmission of BBTV or indeed any other members of the *Nanoviridae*. It has long been considered that phloem-limited viruses are not seed transmitted, owing to the lack of a direct vascular connection to the seed embryo, whereas some mechanically transmissible viruses capable of non-vascular cell-to-cell movement may be seed transmitted (Bennett, 1940). Recent work with begomoviruses, some of which are considered to be phloem-limited, has brought this general assumption into doubt. However, some individual isolates of these viruses, for example tomato leaf curl New Delhi virus, are mechanically transmissible, and in mixed infections with non-transmissible isolates can confer mechanically transmissibility to the latter (Renukadevi et al., 2022). It is unclear how an apparently exclusively phloem-limited virus such as BBTV could reach the seed embryo. The outer integument of the seed, containing the seed’s maternal phloem is isolated from the inner integument, endosperm and embryo, with no symplastic connection. The embryonic suspensor cells are thought to be the route for virus invasion of the embryo, but there is no vascular connection and these symplastic connections are broken prior to fertilization when the suspensor cells undergo programmed cell death (Renukadevi et al., 2022). *C. indica* is partially self-fertile (autogamous), partially cross-pollinated (Sibiya, 2018), leaving open the possibility that infected seeds could also arise through cross-pollination via pollen from a BBTV-infected male parent. However, as with ovule infection, the mechanism that would allow the infection of male gametes with a phloem-limited virus is unclear.

BBTV was detected in the seed coat + endosperm and in the embryo. However, an infected embryo does not always guarantee a virus-infected seedling (Sastry, 2013). Thus, in our work, embryo infection was also supported by PCR detection of BBTV in 28% and 34% of the seedlings from the two seed samples from BBTV-infected *C. indica*, and further by back inoculation of BBTV from infected *C. indica* seedlings to cv. Lakatan banana.

It is probable that seed transmission of BBTV has not been studied or noted before as edible bananas produce parthenocarpic (seedless) fruit. While wild bananas produce seeds, BBTV-infected bananas rarely produce a bunch. Wild bananas have also not been extensively studied with respect to BBTV infection.

This study highlights two alternative means of dispersal of BBTV which may be significant factors in the geographic spread and the maintenance of reservoirs of BBTV: non-*Musa* hosts and seed transmission. BBTV-susceptibility and seed transmission of BBTV in *C. indica* are important concerns to the banana industry, as this plant provides a source of infection through direct aphid transmission and also through vegetative propagation via infected rhizomes or seed.

There are now numerous examples of susceptibility to BBTV in alternative hosts, especially within the order *Zingiberales*. Thus, exploration of other species in the group may reveal additional hosts and advance epidemiology and mitigation studies for BBTV. The findings of this study are significant with the growing demand for ornamentals in the Philippines today. Many growers now propagate ornamental plants at home to avoid a series of quarantine restrictions. Since the wider community is generally not familiar with BBTD, uncontrolled movement of these ornamentals poses a threat to the banana industry. Awareness of the need for responsible propagation and sale of reported alternative host plants must be promoted. Elimination of these species around banana cropping areas may also aid BBTV mitigation measures.

## 5 Conclusion

*M. velutina, M. coccinea, M. laterita, C. indica*, and *C. longa* are alternative hosts of BBTV in the Philippines. These plants may act as reservoirs and sources of the virus in banana growing areas and thus may lessen the efficiency of mitigation practices if they are neglected. Furthermore, BBTV was shown to be seed-borne in the ornamental plant, *Canna indica*. Seed and grow-out seedling samples of naturally and artificially infected plants were positive for BBTV based on PCR assays. BBTV was also transmitted by the aphid vector from seed-transmitted *C. indica* seedlings to banana, demonstrating that *C. indica* is not a “dead-end” host of BBTV and could play a role in the epidemiology of BBTV. The discovery of a new mode of transmission of BBTV is advantageous to mitigation efforts against BBTV. Consideration should be given to the inclusion of the identified alternative hosts in the formulation of quarantine policies regarding the safe movement of the plant hosts. It would be valuable to further explore species belonging to order *Zingiberales* as potential alternative hosts to BBTV, especially crop species grown in banana cropping areas.

## Data Availability

The original contributions presented in the study are included in the article/supplementary material. Further inquiries can be directed to the corresponding author/s.

## Footnotes

^1^ Mendoza, J. S., Dela Cueva, F. M., Nocum, J. D. L., Manohar, A. N. C., Roanne R. Gardoce, R. R., Lachica, G., et al. (in press). A Sensitive Batch Detection of banana bunchy top virus using SYBR Green real-time PCR. Virus Disease.
